# Watervogels – Wintering waterbirds in Flanders, Belgium

**DOI:** 10.3897/zookeys.915.38265

**Published:** 2020-02-24

**Authors:** Koen Devos, Filiep T'jollyn, Peter Desmet, Frederic Piesschaert, Dimitri Brosens

**Affiliations:** 1 Research Institute for Nature and Forest (INBO), Brussels, Belgium Research Institute for Nature and Forest Brussels Belgium; 2 Belgian Biodiversity Platform, Brussels, Belgium Belgian Biodiversity Platform Brussels Belgium

**Keywords:** birds, monitoring, wetlands, population trends, waterbirds, distribution, open data, occurrence, observation

## Abstract

"Watervogels – Wintering waterbirds in Flanders, Belgium" is a sampling event dataset published by the Research Institute for Nature and Forest (INBO). It contains more than 94,000 sampling events (site counts), covering over 710,000 species observations (and zero counts when there is no associated occurrence) and 36 million individual birds for the period 1991–2016. The dataset includes information on 167 different species in nearly 1,100 wetland sites. The aim of these bird counts is to gather information on the size, distribution, and long-term trends of wintering waterbird populations in Flanders. These data are also used to assess the importance of individual sites for waterbirds, using quantitative criteria. Furthermore, the waterbird counts contribute to international monitoring programs, such as the International Waterbird Census (coordinated by Wetlands International) and fulfil some of the objectives of the European Bird Directive, the Ramsar Convention, and the Agreement on the Conservation of African-Eurasian Migratory Waterbirds (AEWA). Here the dataset is published as a standardized Darwin Core Archive and includes for each event: a stable event ID, date and location of observation and a short description of the sampling protocol, effort and conditions (in the event core), supplemented with specific information for each occurrence: a stable occurrence ID, the scientific name and higher classification of the observed species, the number of recorded individuals, and a reference to the observer of the record (in the occurrence extension). Issues with the dataset can be reported at https://github.com/inbo/data-publication/issues.

The following information is not included in this dataset and available upon request: roost site counts, counts from historical (inactive) locations and counts from before 1991.

We have released this dataset to the public domain under a CC0 1.0 Universal (CC0 1.0) Public Domain Dedication (https://creativecommons.org/publicdomain/zero/1.0/). We would appreciate it if you follow the INBO norms for data use (https://www.inbo.be/en/norms-data-use) when using the data. If you have any questions regarding this dataset, do not hesitate to contact us via the contact information provided in the metadata or via opendata@inbo.be.

## Rationale

Counting waterbirds has a long tradition in Flanders, going back to the 1960s. The aim of this long-running monitoring scheme is to gather reliable information on the numbers, trends, and distribution of these species during their winter and migration period. This project provides data for international treaties and conventions such as the European Union (EU) Birds and Habitats Directives, the Ramsar Convention on Wetlands, and the Agreement on the Conservation of African-Eurasian Migratory Waterbirds (AEWA). These results are also used for informed decision-making by conservation bodies, planners and developers, and contribute to the sustainable use and management of wetlands and their dependent waterbirds.

## Waterbirds application

Counts and additional information can be recorded through the waterbirds web application, developed by INBO (http://watervogels.be). This site is only available in Dutch and a login is required. Since 2012, data gathered in the application are available as open data on GBIF. There is a three-year latency between the moment of recording and the moment of data publication, due to quality control and specific agreements with the regional coordinators. The counts are organized with the assistance of Natuurpunt, a regional nature conservation organization. The database is managed by the INBO and is the source for this dataset.

## Taxonomic coverage

**Figure 1.**
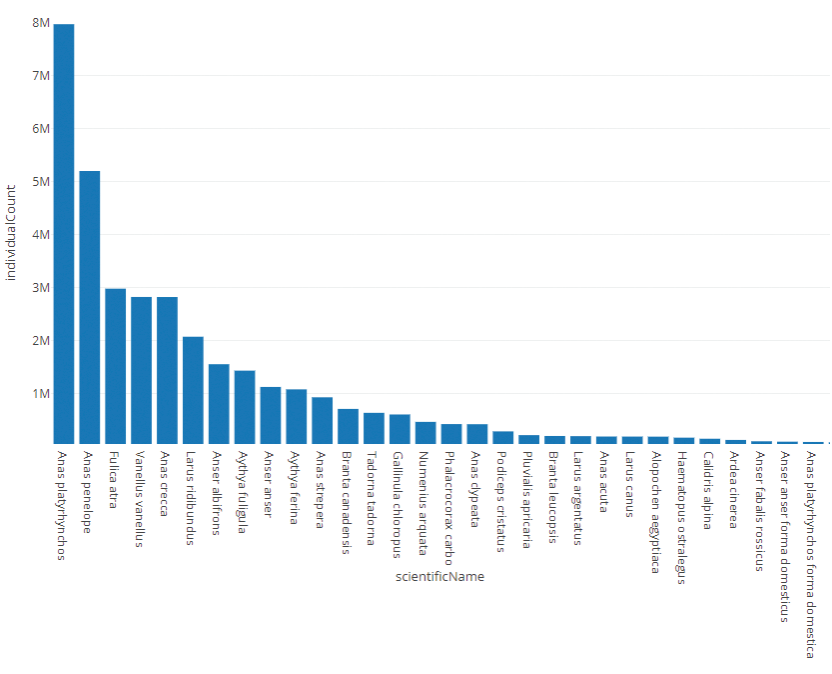
Top 30 most counted (dwc:individualCount) waterbird taxa in Flanders.

The term waterbirds is used as defined in the AEWA and thus does not only include species that belong to the order Anseriformes, but all species which are ecologically dependent on wetlands for at least part of their annual cycle. The dataset includes 167 species (as well as a number of genera, subspecies and forms) belonging to the following species groups: divers, grebes, cormorants, herons, storks, spoonbills, swans, geese, ducks, coots, rails, cranes, waders, gulls, and terns. Non-native species that have been introduced or escaped are also included. The top five of most observed species, collectively representing one third of the dataset, are *Anas platyrhynchos*, *Fulica atra*, *Gallinula chloropus*, *Ardea cinerea*, and *Phalacrocorax carbo*. The 30 most frequently counted (dwc:individualCount) taxa are shown in Figure 1.

For all 183 taxa, the Dutch vernacular name is indicated in vernacularName. To allow interoperability with other databases, the Euring code is added in taxonID for all taxa, except for *Chloephaga melanoptera* which is not listed by Euring.

## Taxonomic ranks

**Kingdom**: Animalia (animals)

**Phylum**: Chordata (chordates)

**Class**: Aves (birds)

**Families**: Alcidae (auks), Anatidae (ducks, geese & swans), Ardeidae (herons), Charadriidae (plovers, dotterels & lapwings), Ciconiidae (storks), Gaviidae (divers), Gruidae (cranes), Haematopodidae (oystercatchers), Laridae (gulls), Pelecanidae (pelicans), Phalacrocoracidae (cormorants), Phoenicopteridae (flamingos), Podicipedidae (grebes), Rallidae (rails), Recurvirostridae (avocets & stilts), Scolopacidae (sandpipers), Stercorariidae (skuas), Threskiornithidae (ibises & spoonbills)

**Species**: *Actitis hypoleucos*, *Aix sponsa*, *Aix galericulata*, *Alle alle*, *Alopochen aegyptiaca*, *Anas formosa*, *Anas penelope*, *Anas carolinensis*, *Anas crecca*, *Anas bahamensis*, *Anas undulata*, *Anas acuta*, *Anas platyrhynchos*, *Anas cyanoptera*, *Anas americana*, *Anas platyrhynchos*, *Anas discors*, *Anas sibilatrix*, *Anas poecilorhyncha*, *Anas strepera*, *Anas capensis*, *Anas falcate*, *Anas rubripes*, *Anas querquedula*, *Anas clypeata*, *Anas flavirostris*, *Anas versicolor*, *Anser anser*, *Anser albifrons*, *Anser fabalis*, *Anser albifrons*, *Anser anser*, *Anser cygnoides*, *Anser indicus*, *Anser erythropus*, *Anser fabalis*, *Anser brachyrhynchus*, *Anser fabalis*, *Ardea alba*, *Ardea purpurea*, *Ardea cinerea*, *Arenaria interpres*, *Aythya marila*, *Aythya nyroca*, *Aythya fuligula*, *Aythya collaris*, *Aythya valisineria*, *Aythya ferina*, *Aythya affinis*, *Botaurus stellaris*, *Branta bernicla*, *Branta sandvicensis*, *Branta leucopsis*, *Branta canadensis*, *Branta hutchinsii*, *Branta bernicla*, *Branta bernicla*, *Branta ruficollis*, *Bubulcus ibis*, *Bucephala albeola*, *Bucephala clangula*, *Cairina moschata*, *Calidris maritima*, *Calidris ferruginea*, *Calidris temminckii*, *Calidris alba*, *Calidris alpine*, *Calidris minuta*, *Calidris melanotos*, *Calidris canutus*, *Callonetta leucophrys*, *Cepphus grylle*, *Charadrius morinellus*, *Charadrius alexandrinus*, *Charadrius hiaticula*, *Charadrius dubius*, *Chen caerulescens*, *Chen canagica*, *Chen rossii*, *Chenonetta jubata*, *Chlidonias hybrida*, *Chlidonias niger*, *Chloephaga picta*, *Chloephaga melanoptera*, *Chroicocephalus ridibundus*, *Ciconia ciconia*, *Clangula hyemalis*, *Crex crex*, *Cygnus atratus*, *Cygnus olor*, *Cygnus cygnus*, *Cygnus columbianus*, *Dendrocygna autumnalis*, *Dendrocygna bicolor*, *Egretta garzetta*, *Eudocimus albus*, *Fulica atra*, *Gallinago gallinago*, *Gallinula chloropus*, *Gavia arctica*, *Gavia stellata*, *Gavia immer*, *Grus grus*, *Haematopus ostralegus*, *Himantopus himantopus*, *Ichthyaetus melanocephalus*, *Ixobrychus minutus*, *Larus hyperboreus*, *Larus fuscus*, *Larus delawarensis*, *Larus michahellis*, *Larus argentatus*, *Larus marinus*, *Larus glaucoides*, *Larus canus*, *Larus cachinnans*, *Larus minutus*, *Limosa lapponica*, *Limosa limosa*, *Lophodytes cucullatus*, *Lymnocryptes minimus*, *Marmaronetta angustirostris*, *Melanitta nigra*, *Melanitta fusca*, *Mergellus albellus*, *Mergus serrator*, *Mergus merganser*, *Netta rufina*, *Numenius arquata*, *Numenius phaeopus*, *Nycticorax nycticorax*, *Oxyura leucocephala*, *Oxyura jamaicensis*, *Pelecanus rufescens*, *Pelecanus onocrotalus*, *Phalacrocorax aristotelis*, *Phalacrocorax carbo*, *Phalaropus lobatus*, *Phalaropus fulicarius*, *Philomachus pugnax*, *Phoeniconaias minor*, *Phoenicopterus ruber*, *Platalea leucorodia*, *Plegadis falcinellus*, *Pluvialis fulva*, *Pluvialis apricaria*, *Pluvialis squatarola*, *Podiceps nigricollis*, *Podiceps cristatus*, *Podiceps auritus*, *Podiceps grisegena*, *Porzana porzana*, *Rallus aquaticus*, *Recurvirostra avosetta*, *Rissa tridactyla*, *Scolopax rusticola*, *Somateria mollissima*, *Stercorarius parasiticus*, *Stercorarius pomarinus*, *Stercorarius skua*, *Sterna paradisaea*, *Sterna hirundo*, *Sternula albifrons*, *Tachybaptus ruficollis*, *Tadorna cana*, *Tadorna ferruginea*, *Tadorna tadorna*, *Thalasseus sandvicensis*, *Threskiornis aethiopicus*, *Tringa ochropus*, *Tringa erythropus*, *Tringa totanus*, *Tringa glareola*, *Tringa nebularia*, *Uria aalge*, *Vanellus gregarius*, *Vanellus vanellus*, *Xema sabini*.

## Geographic coverage

**Figure 2.**
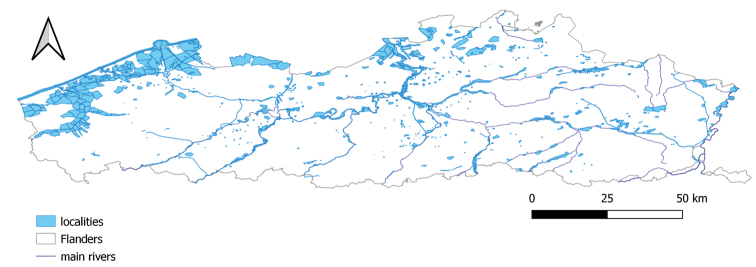
Map of the waterVogelTelgebieden (localities) in Flanders in which the waterbirds are counted. The geospatial dataset is available in this repository.

The birds were counted at 1,189 predefined locations (waterVogelTelgebieden, Figure 2), with a total surface of 141,000 ha and covering a large part of wetland and coastal habitats in Flanders, Belgium. These locations are visited regularly during the wintering and migration season (mid-monthly, from October to March). For each event, the code for the waterVogelTelgebied is indicated in locationID. Birds counted at sea are not included in this dataset.

## Bounding box

50.68° to 51.51° latitude, 2.54° to 5.92° longitude

## Temporal coverage

1991-01-08 to 2016-03-24

## Methodology

### Study extent description

The bird counts are organized as a regional network "Waterbird counts Flanders" coordinated by the Research Institute for Nature and Forest (INBO). The network divides Flanders, Belgium into 24 regions, each of them with a local coordinator. The fieldwork is mainly done by skilled volunteer birdwatchers, often working together within local bird clubs. The NGO Natuurpunt (http://natuurpunt.be) supports the majority of these bird clubs and volunteers, and thereby delivers an important contribution to the waterbird project. A number of large and important wetland areas are counted by INBO staff (especially in the Scheldt estuary and along the Yser River).

To allow reliable comparisons between years and between areas, the counts are aimed for maximum standardization. Every winter, birds are counted in six monthly censuses from October to March, on the weekend the closest to the 15^th^ of the month. Counts use the same methodology and are made at more than 1,100 predefined locations (see Geographic coverage), covering all kinds of wetland habitats in Flanders, such as lakes, ponds, reservoirs, and rivers. They also include agricultural areas, often holding large numbers of waterbirds (such as wintering geese). Although the project aims for a (nearly) complete coverage of all areas hosting substantial numbers of waterbirds, this is difficult to achieve and the number of counted sites varies between months and years.

During counts, numbers of all waterbird species are recorded. This includes divers, grebes, cormorants, herons and allies, swans, geese, ducks, coots and rails. Waders and gulls (optional) have been added to the species list in 1999. Counts of coastal waders are, however, available since 1992.

Through the watervogels web application, http://watervogels.inbo.be, volunteers can enter their count and additional data directly into a central database.

### Sampling description

The counts are done at predefined locations, called waterVogelTelgebied. The name of each waterVogelTelgebied is indicated in locality, while its ID is indicated in locationID. The polygon shape for most of these localities can be found at https://tinyurl.com/t5tdt6v. The geographic coordinates for the occurrences represent the centroid of the locality.

Within the borders of these areas, present waterbird species are counted as completely as possible. Clearly visible areas are often counted from one point with a telescope. Large and less visible areas are usually traversed on foot, by bicycle or by car. A special case are the monthly counts on the Zeeschelde which are performed from boats by INBO staff. The count method (e.g., survey on land) is indicated in samplingProtocol, while the achieved effort and the completeness of the survey (e.g. complete survey of location & all waterbirds counted) is indicated in samplingEffort. In dynamicProperties, we provide the sample conditions in JSON format:

(e.g., {"samplingConditions":"favourable", "samplingCoverage":"complete", "snow":"none", "ice":"0%", "waterLevel":"normal"})

To reduce the likelihood of birds being double counted or missed, the counts are synchronized as much as possible. Counts are organized on the weekend closest to the 15^th^ of the month. In large areas with a high probability of local movements, observers are asked to pay special attention to count more or less simultaneously, preferably with multiple observers. Birds are counted during daytime, while specific high tide counts are organized for typical coastal waders (gathering on specific high tide roosts). The Zeeschelde on the contrary, is mainly counted at low tide due to better visibility of the birds. For some species that are dispersed widely during the day, simultaneous counts on the roost sites are a better alternative for gathering information on their population size. Each winter, supplementary counts are organized for Great Cormorant (since 2003), gulls and Eurasian Curlew. These roost counts are however not included in this database.

Keep in mind that covering of the sites differs between months and years. Calculations of trends and population sizes therefore have to deal with missing values.

Quality control description

All published records are validated. The initial validation is done manually by the INBO waterbird expert. If, during the analysis of the data, outliers are found, these records will also be removed from the database.

## Method step description

These are the steps for entering data into the centralized database:

1. Indicate date, start time, and end time (all expressed in eventDate).

2. Indicate observer (recordedBy).

3. Indicate specific count area (locality). The area has a unique ID (locationID) and is linked higher geography (continent & countryCode). Together with the time information, this constitutes a count, which has a unique ID as well (eventID).

4. Indicate count method (samplingProtocol) and achieved effort (samplingEffort).

5. Indicate the count conditions, such as samplingConditions, samplingCoverage, snow, ice, and waterLevel (all expressed as json in dynamicProperties).

6. For each observed waterbird species (scientificName), indicate the estimated number of birds (individualCount).

For publication, the data is further processed:

1. Each record gets a GUID, based on the ID assigned by the database (occurrenceID).

2. The locationID is cross referenced with the geospatial information for the localities (https://tinyurl.com/t5tdt6v).

3. Taxonomy information is added based on the scientific name and expressed in kingdom, phylum, class, taxonRank, nomenclaturalCode, and scientificNameAuthorship, as well as an Euring code (taxonID) and Dutch vernacular name (vernacularName).

4. Dataset metadata information is added (type, basisOfRecord, language, datasetID, datasetName, institutionCode) as well the rights holder (rightsHolder), the license (rights) and data use norms (accessRights).

## Dataset

### Dataset description

The Darwin Core terms (http://rs.tdwg.org/dwc/terms/) in the dataset are: occurrenceID, type, language, license, rightsHolder, accessRights, datasetID, institutionCode, datasetName, dynamicProperties, basisOfRecord, recordedBy, individualCount, eventID, samplingProtocol, samplingEffort, eventDate, locationID, continent, countryCode, locality, decimalLatitude, decimalLongitude, geodeticDatum, georeferenceRemarks, taxonID, scientificName, kingdom, phylum, class, taxonRank, scientificNameAuthorship, vernacularName, and nomenclaturalCode.

**Object name**: Watervogels - Wintering waterbirds in Flanders, Belgium

**Format name**: Darwin Core Archive format

**Format version**: 1.0

**Character encoding**: UTF-8

**Language**: English

**License**: http://creativecommons.org/publicdomain/zero/1.0/

**Usage norms**: http://www.inbo.be/en/norms-for-data-use

**Publication date**: 2019-12-16

**Distribution**: https://ipt.inbo.be/resource?r=watervogels-occurrences

**DOI**: https://doi.org/10.15468/lj0udq

**Version**: 3.8

### Data records

The data are standardized to Darwin Core (Wieczorek et al. 2012) with a custom SQL view on the INBO ‘watervogels’ database. They are published using the GBIF Integrated Publishing Toolkit (Robertson et al. 2014) instance at the INBO (https://ipt.inbo.be).

The data are organized as a sampling event resource, with an event core containing 94,163 records and 1 occurrence extension containing 717,006 records.

The INBO IPT archives the data and thus serves as the data repository. The data and resource metadata are available for download in the downloads section. The versions table lists other versions of the resource that have been made publicly available and allows tracking changes made to the resource over time.

### Additional information

The following information is not included in this dataset and available upon request: roost site counts, counts from historical (inactive) locations.

**Dataset**

**Project title**

Waterbird counts Flanders

**Funding**

This monitoring project receives funding from the Flemish Government.

The Waterbirds application is developed with financial support from the EU (LIFE14 IPE BE 002 Belgian Nature Integrated Project)

**Content providers**

Many volunteers from Natuurpunt.

## References

[B1] AEWA [Agreement on the Conservation of African-Eurasian Migratory Waterbirds] (1995) Art. 3, 16 June 1995, 2365 UNTS 251. http://www.unep-aewa.org/

[B2] Directive 2009/147/EC (2009) Directive 2009/147/EC of the European Parliament and of the Council of 30 November 2009 on the conservation of wild birds. Special Edition in Croatian 032(15): 128–146. http://data.europa.eu/eli/dir/2009/147/oj

[B3] EURING [The European Union for Bird Ringing] (2010) The EURING Exchange Code 2000 Plus. Online Code Tables. Thetford, UK. http://www.euring.org/data_and_codes/euring_code_list/index.html

[B4] Habitats Directive (1992) Habitats Directive: Council Directive 92/43/EEC of 21 May 1992 on the conservation of natural habitats and of wild fauna and flora. http://data.europa.eu/eli/dir/1992/43/2013-07-01

[B5] Ramsar Convention (2005) A conceptual framework for the wise use of wetlands and the maintenance of their ecological character. Resolution 9.1 Annex A. http://www.ramsar.org/sites/default/files/documents/pdf/res/key_res_ix_01_annexa_e.pdf

[B6] Robertson T Doring M Guralnick R Bloom D Wieczorek J Braak K Otegui J Russell L Desmet P (2014) The GBIF Integrated Publishing Toolkit: Facilitating the Efficient Publishing of Biodiversity Data on the Internet. PLoS ONE 9: e102623. 10.1371/journal.pone.0102623 PMC4123864 25099149

[B7] Wieczorek J Bloom D Guralnick R Blum S Doring M Giovanni R Robertson T Vieglais D (2012) Darwin Core: An Evolving Community-Developed Biodiversity Data Standard. PLoS ONE 7: e29715. 10.1371/journal.pone.0029715 PMC3253084 22238640

